# Ubiquitin Ligase Huwe1 Modulates Spermatogenesis by Regulating Spermatogonial Differentiation and Entry into Meiosis

**DOI:** 10.1038/s41598-017-17902-0

**Published:** 2017-12-19

**Authors:** Rohini Bose, Kai Sheng, Adel R. Moawad, Gurpreet Manku, Cristian O’Flaherty, Teruko Taketo, Martine Culty, Kin Lam Fok, Simon S. Wing

**Affiliations:** 1 The Research Institute of the McGill University Health Centre, Montréal, Québec Canada; 2 Department of Medicine, McGill University, Montréal, Québec Canada; 3 Department of Surgery, McGill University, Montréal, Québec Canada; 4 Department of Pharmacology and Therapeutics, McGill University, Montréal, Québec Canada; 5 Department of Obstetrics and Gynecology, McGill University, Montréal, Québec Canada; 6 Department of Biology, McGill University, Montréal, Québec, Canada; 7 Department of Theriogenology, Faculty of Veterinary Medicine, Cairo University, Giza, Egypt; 8 Present Address: Dept. of Pharmacology, University of Southern California, Los Angeles, California USA; 9 Epithelial Cell Biology Research Center, School of Biomedical Sciences, The Faculty of Medicine, The Chinese University of Hong Kong, Sha Tin, Hong Kong; 10 Shenzhen Research Institute, The Chinese University of Hong Kong - Shenzhen, Shenzhen, China

## Abstract

Spermatogenesis consists of a series of highly regulated processes that include mitotic proliferation, meiosis and cellular remodeling. Although alterations in gene expression are well known to modulate spermatogenesis, posttranscriptional mechanisms are less well defined. The ubiquitin proteasome system plays a significant role in protein turnover and may be involved in these posttranscriptional mechanisms. We previously identified ubiquitin ligase Huwe1 in the testis and showed that it can ubiquitinate histones. Since modulation of histones is important at many steps in spermatogenesis, we performed a complete characterization of the functions of Huwe1 in this process by examining the effects of its inactivation in the differentiating spermatogonia, spermatocytes and spermatids. Inactivation of Huwe1 in differentiating spermatogonia led to their depletion and formation of fewer pre-leptotene spermatocytes. The cell degeneration was associated with an accumulation of DNA damage response protein γH2AX, impaired downstream signalling and apoptosis. Inactivation of Huwe1 in spermatocytes indicated that Huwe1 is not essential for meiosis and spermiogenesis, but can result in accumulation of γH2AX. Collectively, these results provide a comprehensive survey of the functions of Huwe1 in spermatogenesis and reveal Huwe1’s critical role as a modulator of the DNA damage response pathway in the earliest steps of spermatogonial differentiation.

## Introduction

Spermatogenesis is a tightly regulated process that ensures the successful production of millions of genetically unique haploid spermatozoa each day. Occurring within the seminiferous epithelium in tubules of the testis, spermatogenesis is sustained by a small population of undifferentiated spermatogonial progenitors which include the spermatogonial stem cells^1^. Throughout adulthood, some of these progenitors become committed to differentiation. This initiation of differentiation takes place asynchronously throughout the testis to assure continuous production of sperm. However, at any specific location in the seminiferous epithelium, differentiation is initiated in a precisely timed manner, which in the mouse occurs every 8.6 days. Retinoic acid, through the induction of the *Stra8* gene, is the critical signal for inducing this transition to A_1_ spermatogonia, the earliest differentiated spermatogonia^2^. Subsequently, the A_1_ spermatogonia proceed through six rounds of synchronous cell divisions in rodents (A_2_, A_3_, A_4_, Intermediate (In), Type B spermatogonia) to finally give rise to primary or pre-leptotene spermatocytes^3,4^ in early meiosis. During meiotic prophase I, homologous chromosomal recombination occurs which is critical for creating the genetically diverse spermatids. The induction of *Spo11* recombinase plays a vital role in the generation of double strand breaks in this process^5^. Following the two rounds of meiotic division, the haploid spermatids undergo a process of cellular remodeling and condensation, referred to as spermiogenesis. As part of this process, histones are removed and replaced by protamines, which enable tighter packing of the chromatin^6^. Defects in spermatogenesis lead to azoospermia in humans^7,8^ and so it is important to acquire a comprehensive understanding of the molecular mechanisms underlying spermatogonial differentiation. There are plentiful studies describing the transcriptional programs controlling spermatogonial differentiation^9–15^. However, the post-transcriptional regulation underlying spermatogonial differentiation remains largely unknown.

The ubiquitin system has both proteolytic and non-proteolytic functions in spermatogenesis^16^. The system functions by activating and transferring ubiquitin to proteins by a three-enzyme cascade: the E1 or the ubiquitin-activating enzyme, E2 or the ubiquitin-conjugating enzyme and E3 or the ubiquitin ligase. Ubiquitin ligases are responsible for substrate selectivity and specificity and mediate the final step of covalently tagging proteins with the ubiquitin moiety. Huwe1 is a ubiquitin ligase that was first identified by our laboratory as a testis E3 that can ubiquitinate all core histones *in vitro* ^17^. Since regulation of histones plays an important role in many steps of spermatogenesis, we inactivated the enzyme specifically in the germ cells to characterize its functions. Inactivation of Huwe1 in the gonocytes leads to a defect in establishment and maintenance of spermatogonia resulting in a Sertoli cell-only phenotype in the adult testis and infertility^18^. Cell degeneration in the knockout testis occurs through mitotic catastrophe associated with a hyperactivation of the DNA damage response pathway (DDR) attributed to the accumulation of both histone H2AX and its phosphorylated isoform γH2AX in undifferentiated spermatogonia^18^.

Since loss of Huwe1 in gonocytes results in depletion of spermatogonia starting as early as 6 days post partum (dpp), we were unable to study functions of the ligase beyond this point. To explore for additional roles of Huwe1, we inactivated the enzyme using Cre-recombinase driven by the *Stra8* promoter which turns on in type A_1_ spermatogonia and is active up to the pre-leptotene spermatocytes^19^ and by the *Spo11* promoter which is activated in spermatocytes that have initiated meiosis^20^. With this approach, we show that indeed Huwe1 has critical roles in spermatogonial differentiation leading up to meiosis.

## Results

### Inactivation of Huwe1 in differentiating spermatogonia leads to an arrest in spermatogenesis

**Figure 1:**
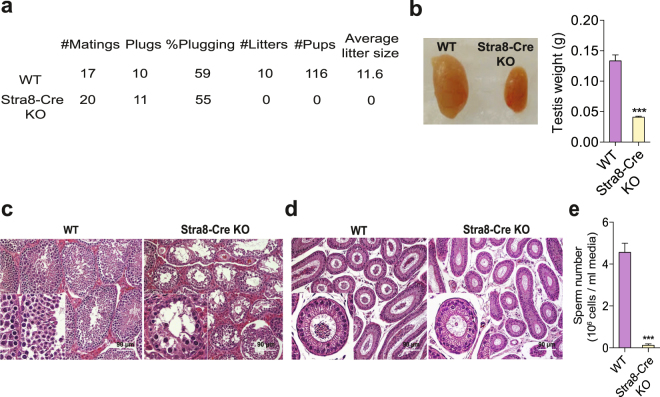
Inactivation of Huwe1 in the differentiating spermatogonia leads to an arrest in spermatogenesis. (**a**) Inactivation of Huwe1 in the differentiating spermatogonia leads to infertility. WT and Stra8-Cre KO (n = 5) male mice were mated with CD1 females for 4 consecutive weeks. The females were changed every week. Indicated are the frequency of vaginal plugging, number of litters sired and the average litter size. (**b**)Adult Stra8-Cre KO mice have smaller testes than WT mice. Representative images (left panel) and weights (right panel) of testis from WT (n = 9) and Stra8-Cre KO (n = 10) mice. (**c**) Arrested spermatogenesis in the Stra8-Cre KO testis. Hematoxylin and eosin stained testicular tissue sections from adult (4 month old) WT and Stra8-Cre KO testes. Scale bar = 90 μm. (**d**) Few sperm in the Stra8-Cre KO epididymis. Hematoxylin and eosin stained caput (head of the epididymis) tissue sections from adult WT and Stra8-Cre KO. Scale bar = 90 μm. (**e**) Low sperm number in the Stra8-Cre KO mice. Measurement of sperm number in the cauda (tail of the epididymis) of WT and Stra8-Cre KO (n = 5) mice. Data are shown as mean ± SEM. Student’s t-test. ***p < 0.001.

To identify roles for Huwe1 in germ cells committed to the spermatogenic program, we inactivated Huwe1 in the differentiating spermatogonia by crossing conditional *Huwe1* knockout female mice (*Huwe1* ^*flox/flox*^)^21^ with males expressing a *Stra8-Cre* transgene hemizygously^19^. The *Stra8* gene is activated directly by retinoic acid and is one of the earliest genes induced in differentiating spermatogonia and therefore a marker of A1 spermatogonia^22^. Since *Huwe1* is an X-linked gene, all the males obtained from this cross were either the genotype *Huwe1* ^*flox/Y*^ (hereafter referred to as WT) or *Huwe* ^*−/Y*^ *Stra8-Cre* (hereafter referred to as Stra8-Cre KO). Excision of the lox-P flanked region was confirmed by RT-PCR of whole testes RNA (Supplementary Figure 1). A fertility assay comparing the two genotypes revealed that the Stra8-Cre KO males had similar copulatory behavior, measured as the frequency of vaginal plugging (Fig. 1a). However, they were unable to sire any litters. In addition, the average weight of their adult testes was only 30% of the WT (Fig. 1b). Examination of histological sections of testes revealed that there was significant loss of differentiating spermatogonia, spermatocytes and extensive loss of spermatids in the tubules (Fig. 1c). The epididymis of the Stra8-Cre KO had only a few sperm (Fig. 1d) with the average caudal sperm number being 3% of that seen in the WT (Fig. 1e). Collectively, these results indicate that Huwe1 activity in the differentiating spermatogonial population is crucial for male fertility, its inactivation leading to a developmental arrest early in spermatogenesis.

### Huwe1 is a critical regulator of spermatogonial differentiation

**Figure 2:**
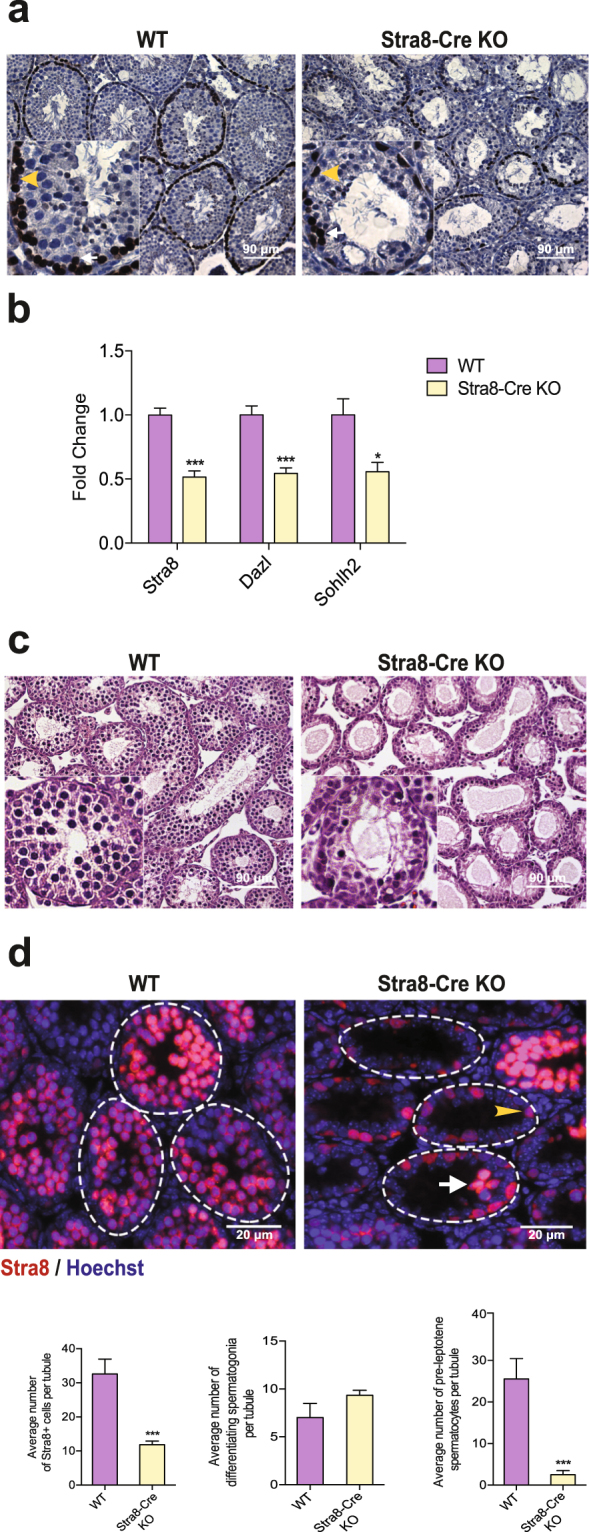
Huwe1 is a critical regulator of spermatogonial differentiation. (**a**) Disrupted formation of pre-leptotene spermatocytes in synchronized Stra8-Cre KO testis 48 days post retinoic acid (dpRA) adult testes. Representative immunohistochemical images from WT and Stra8-Cre KO testis sections stained for Stra8. Pre-leptotene spermatocytes are identified based on their Stra8 positivity and location in the seminiferous tubules away from the basement membrane. The yellow arrowhead indicates an A_1_ differentiating spermatogonia and the white arrow indicates a pre-leptotene spermatocyte. Scale bar = 90 μm. (**b**) Expression of markers for differentiating spermatogonia is decreased in synchronized Stra8-Cre KO 48 dpRA adult testes. Q-PCR analysis of markers of differentiating spermatogonia (*Stra8*, *Dazl, Sohlh2*) in the WT (n = 5) and Stra8-Cre KO (n = 6) testis. (**c**) Loss of pre-leptotene/leptotene spermatocytes (germ cells located away from the basement membrane) in synchronized Stra8-Cre KO 8 dpRA tubules. Hematoxylin and eosin stained testicular tissue sections from neonatal WT and Stra8-Cre KO mice. Scale bar = 90 μm. (**d**) Few pre-leptotene spermatocytes in the synchronized Stra8-Cre KO 8 dpRA testis. Representative immunofluorescence images of synchronized WT and Stra8-Cre KO testis stained for Stra8 (red) (top panel). Stra8 + cells at the basement membrane of tubule are A_1_ differentiating spermatogonia (arrowhead); Stra8 + cells away from the basement membrane are pre-leptotene spermatocytes (arrow). Quantification from the images (bottom panel). WT, n = 5 (135 tubules) Stra8-Cre KO, n = 7 (271 tubules). Scale bar = 20 μm. Data are shown as mean ± SEM. Student’s t-test. ***p < 0.001, *p < 0.05.

During spermatogenesis, seminiferous tubules transition through multiple stages, each constituting different sets of cellular associations. As this process occurs asynchronously amongst tubules, a typical cross section of a testis will present tubules in different stages of spermatogenesis. Since staging of tubules depends on analysis of structural features of the acrosome, nucleus and position of spermatids, its accuracy is limited in scenarios such as the Stra8-Cre KO testis where spermatids are absent. To sharpen the resolution of stage-specific defects occurring in the KO testis, we synchronized the tubules to ensure simultaneous division and differentiation of the germ cells with a strategy previously described by Hogarth *et al*.^23^. WT and Stra8-Cre KO mice were fed with WIN 18,446, an inhibitor of retinaldehyde dehydrogenase, an enzyme that is essential for retinoic acid (RA) synthesis. This treatment resulted in the accumulation of undifferentiated spermatogonia. The mice were then given an injection of retinoic acid to initiate differentiation simultaneously in all the tubules. The animals were sacrificed 48 days post RA injection (dpRA), a time-point at which we expected the tubules to have completed the first wave of spermatogenesis and be in Stage VIII containing specific cell types namely, the A_s_, A_pr_ (undifferentiated spermatogonia), A_1_ spermatogonia, pre-leptotene and pachytene spermatocytes and step 8 and 16 spermatids. The testis sections were stained with antibody against Stra8 (expressed from A_1_ spermatogonia to pre-leptotene spermatocytes) to identify the spermatogonia (located adjacent to the basement membrane of the tubules) and the pre-leptotene spermatocytes (located away from the basement membrane)^22^ (Fig. 2a). The Stra8-Cre KO testis had fewer Stra8^+^ pre-leptotene spermatocytes, which indicated that there was a defect in the process of spermatogonial differentiation. This was confirmed by measuring the expression of markers of spermatogonial differentiation (*Stra8, Dazl, Sohlh2*) by Q-PCR, which were markedly decreased in the Stra8-Cre KO testis (Fig. 2b).

Since these results suggested a defect in spermatogonial differentiation, we focused on this process by sacrificing the animals at 8 dpRA when the WT testis tubules will contain either pre-leptotene or leptotene spermatocytes from the first round of differentiation or A_1_ or A_2_ spermatogonia from the second round of differentiation. Hematoxylin and eosin staining of these sections showed a clear loss of pre-leptotene/leptotene spermatocytes (germ cells located away from the basement membrane) in the tubules of the Stra8-Cre KO testis (Fig. 2c). To identify the germ cells more precisely, we stained the sections with antibody against Stra8, which labels spermatogonia and pre-leptotene spermatocytes, but not leptotene spermatocytes (Fig. 2d). There was a significant decrease in the average number of Stra8 + cells in the Stra8-Cre KO testis. While there was no difference in spermatogonia (Stra8 + cells located adjacent to the basement membrane of the tubules), the number of pre-leptotene spermatocytes (Stra8 + cells located away from the basement membrane of the tubules) was significantly lower in the Stra8-Cre KO testis (Fig. 2d). Taken together, these results show that the inactivation of Huwe1 leads to disrupted spermatogonial differentiation and hence perturbed formation of pre-leptotene spermatocytes.

### Inactivation of Huwe1 leads to a defect in entry into meiosis

**Figure 3:**
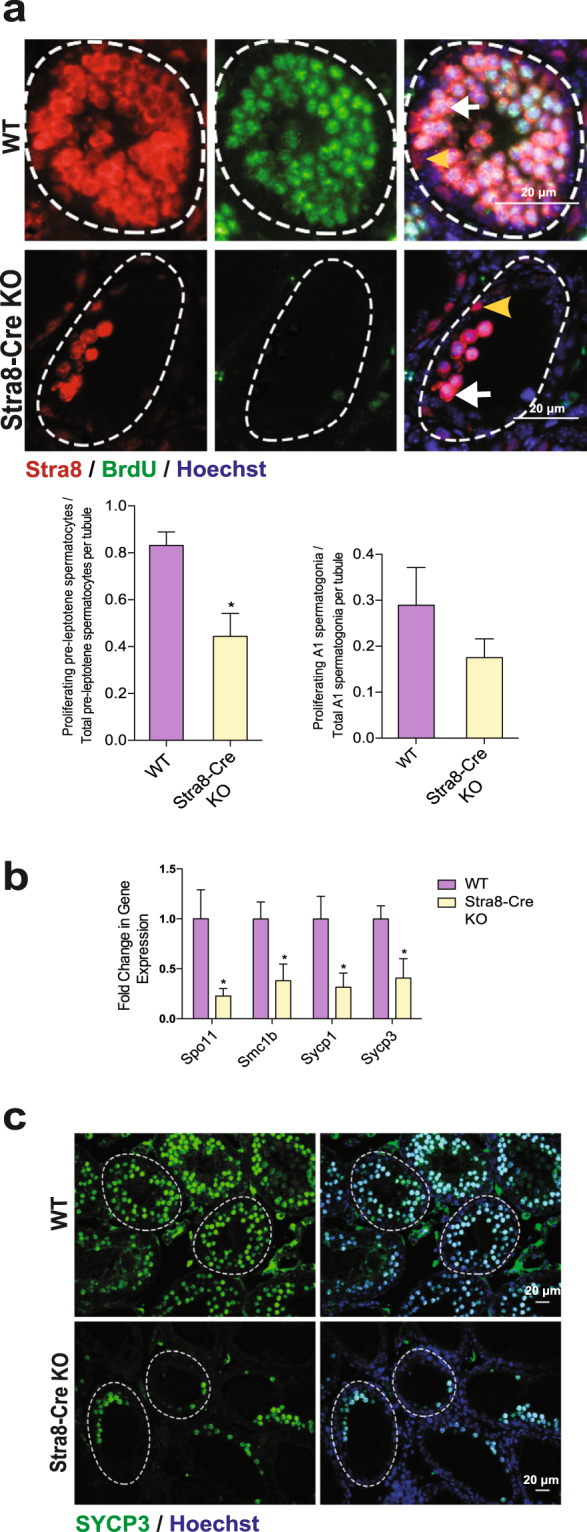
Huwe1 is important for entry into meiosis. (**a**) Inactivation of Huwe1 in the differentiating spermatogonia leads to a defect in proliferation. Representative immunofluorescence images of synchronized 8 dpRA WT and Stra8-Cre KO testis stained for Stra8 (red) and BrdU (green) (top panel). Stra8 + cells at the basement membrane of tubule are A_1_ differentiating spermatogonia; Stra8 + cells away from the basement membrane are pre-leptotene spermatocytes. The yellow arrowhead indicates an A_1_ differentiating spermatogonium and the white arrow indicates a pre-leptotene spermatocyte. Quantification from these images (bottom panel) WT, n = 5 (135 tubules), Stra8-Cre KO, n = 7 (271 tubules). Scale bar = 20 μm. (**b**) Expression of meiotic markers is decreased in the Stra8-Cre KO 8 dpRA testes. Q-PCR analysis of markers of meiosis (*Spo11, Smc1b, Sycp1, Sycp3*) on WT and Stra8-Cre KO 8 dpRA testes (WT, n = 5, Stra8-Cre KO, n = 6). (**c**) Disrupted formation of leptotene spermatocytes in Stra8-Cre KO 8 dpRA testes. Representative immunofluorescence images from WT and Stra8-Cre KO testis sections stained for SYCP3 (green). Scale bar = 20 μm. Data are shown as mean ± SEM. Student’s t-test. *p < 0.05.

Pre-leptotene spermatocytes undergo a single round of DNA replication, an essential process for the subsequent reductive division occurring in prophase I of meiosis^24^. Since recent work in our laboratory has shown Huwe1 to be important for the mitotic reentry of gonocytes^18^, we asked if it regulates this developmental transition as well. To this end, we injected BrdU at 7 dpRA and sacrificed the mice 24 hr later. Immunofluorescence staining for BrdU and Stra8 was performed to identify the proliferating differentiating spermatogonia and pre-leptotene spermatocytes (Fig. 3a). Quantification revealed that the proportion of pre-leptotene spermatocytes incorporating BrdU was approximately 50% lower in the Stra8-Cre KO testis compared to the WT. There was no significant difference in the proportion of differentiating spermatogonia that were incorporating BrdU between the two groups (Fig. 3a). In addition, qPCR measurement of markers of early meiosis (*Spo11, Smc1b, Sycp1, Sycp3*) revealed a significant down-regulation of the expression of these genes (Fig. 3b). Staining with SYCP3 (a commonly used marker to identify different types of spermatocytes in meiotic prophase I)^25^ confirmed that the Stra8-Cre KO testis had fewer leptotene spermatocytes (Fig. 3c). Taken together, these experiments revealed Huwe1’s critical role as a regulator of proliferation during commitment to meiosis.

### Inactivation of Huwe1 in the differentiating spermatogonia leads to an activation of the DDR and cell death through apoptosis

**Figure 4:**
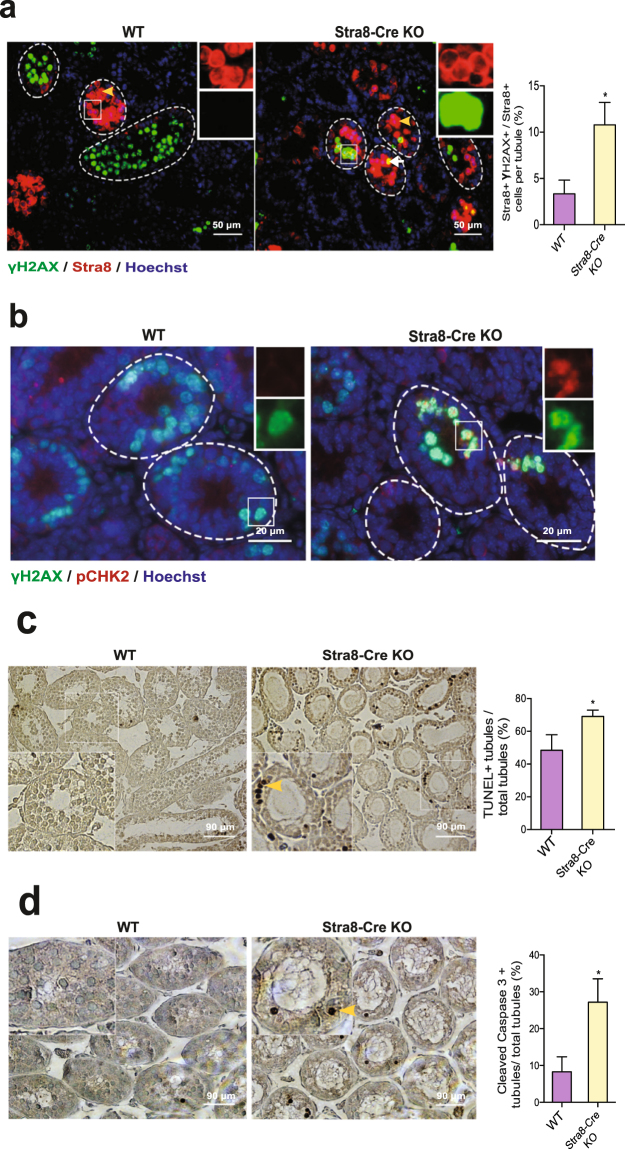
Inactivation of Huwe1 in the differentiating spermatogonia leads to an activation of DDR and apoptotic cell death. (**a**) Loss of Huwe1 resulted in elevated proportion of Stra8 + cells showing intense γH2AX foci in unsynchronized 10 dpp testis. Representative immunofluorescence images from unsynchronized 10 dpp WT and Stra8-Cre KO testes stained for γH2AX (green) and Stra8 (red) (left panel). γH2AX+ Stra8- cells in the tubules are germ cells in early meiosis (leptotene/zygotene). The yellow arrowheads indicate Stra8 + differentiating spermatogonia/pre-leptotene spermatocytes and the white arrow indicates a Stra8+ cell that is also γH2AX+ . Quantification of the proportion of Stra8+ cells that are γH2AX + from the images (right panel) WT, n = 4 (89 tubules), KO, n = 4 (128 tubules). Scale bar = 50 μm. (**b**) Inactivation of Huwe1 leads to the recruitment of pCHK2 to the intense γH2AX foci in unsynchronized 10 dpp testis. Representative images from immunofluorescence staining of 10 dpp WT and Stra8-Cre KO testes stained for γH2AX (green) and pCHK2 (red). Scale bar = 20 μm. (**c**) Loss of Huwe1 in the differentiating spermatogonia resulted in increased levels of apoptosis in the synchronized 8 dpRA testes. Shown are typical images from TUNEL assay performed on testes sections at 8 dpRA (left panel). The yellow arrowheads indicate apoptotic cells. Scale bar = 90 μm. Quantification from the images (right panel). WT, n = 5 (783 tubules) Stra8-Cre KO, n = 7 (1099 tubules). (**d**) Loss of Huwe1 resulted in an elevated percentage of cleaved caspase 3 positive tubules in the synchronized 8 dpRA testes. Shown are typical IHC images for cleaved caspase 3 in 8 dpRA WT and Stra8-Cre KO testes (left panel). The yellow arrowhead indicates a cleaved caspase 3 positive cell. Scale bar = 90 μm. Quantification from the images (right panel) WT, n = 4 (269 tubules), KO, n = 4 (286 tubules). Data are shown as mean ± SEM. Student’s t-test. *p < 0.05.

Our previous studies on the role of Huwe1 in undifferentiated spermatogonia showed that the enzyme is important for regulating H2AX levels. Huwe1 deficiency in these cells leads to elevated H2AX and a hyperactivation of the DDR resulting in mitotic catastrophe^18^. We therefore asked if Huwe1 performs a similar function during the process of spermatogonial differentiation. To this end, we isolated testes from WT and Stra8-Cre KO mice at 10 dpp, a time point at which we expected to capture all the differentiating spermatogonia and pre-leptotene spermatocytes with the most advanced cell type being the leptotene spermatocytes^26^. We stained the sections for γH2AX and Stra8 (Fig. 4a) and found an increased proportion of differentiating spermatogonia and pre-leptotene spermatocytes in the Stra8-Cre KO testis showing intense γH2AX foci. Activation of the ATM arm of the DDR pathway, upstream of H2AX, was further confirmed by staining for another of its targets, pCHK2 (Fig. 4b). To examine events downstream of the recruitment of γH2AX, we stained for ubiquitinated foci which reflects propagation of the DDR. Surprisingly, the excess clustering of γH2AX did not lead to significant staining for ubiquitin (Supplementary Figure 3) of the γH2AX foci, suggesting a defect in the DDR immediately downstream of γH2AX in the KO cells. Testes from 6 dpp mice where Huwe1 had been inactivated using Cre recombinase driven by Dxd4 promoter, were used as a positive control for observing the accumulation of ubiquitinated foci^18^. Although the inactivation of Huwe1 in gonocytes does not lead to increased apoptosis but death via mitotic catastrophe, the DDR is well recognized to promote apoptosis in other cell contexts^27^. Therefore, we explored whether the activation of the DDR pathway in the Stra8-Cre KO testis led to increased levels of apoptosis. Indeed, we found that there was a significant increase in apoptosis in the 8 dpRA Stra8-Cre KO testis as measured by the TUNEL assay of DNA fragmentation (Fig. 4c). We confirmed this result by staining the same sections with cleaved, activated caspase 3 (Fig. 4d), a downstream effector enzyme in the apoptotic cascade^28,29^. Co-staining for Tra98 (a germ cell marker) and cleaved caspase 3 showed that this increase in apoptosis in the Stra8-Cre KO tubules was not due to an increase in apoptosis of Sertoli cells, as approximately 68% of the apoptotic cells in the Stra8-Cre KO tubules were germ cells compared to 46% in the WT, as determined by double staining for Tra98 (a germ cell marker) and cleaved caspase 3 (data not shown). To explore whether this apoptosis was mediated through activation of p53, we stained the testis sections with anti-phosphorylated p53 antibody. There was no significant colocalization of p53 to the nuclei of cells that accumulated γH2AX (Supplementary Figure 4). As a positive control, sections from normal testis that had been irradiated were analyzed in parallel and in contrast, these demonstrated robust co-localization of p53 with γH2AX. From these findings, it may be concluded that the inactivation of Huwe1 in the differentiating spermatogonia led to an activation of the DDR and death through p53-independent apoptosis, thus preventing their progression into the meiotic program.

### Huwe1 is not essential for meiotic progression

**Figure 5:**
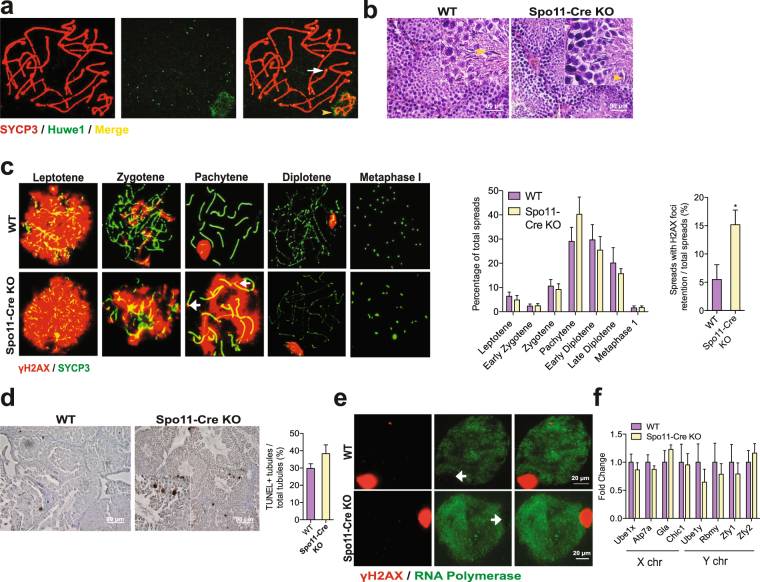
Huwe1 is not essential for completion of the later stages of meiosis. (**a**) Huwe1 is localized to the sex body and telomeres in pachytene spermatocytes. Representative immunofluorescence image from a chromosome spread prepared from a WT testis at 28 dpp stained for Huwe1 (green) and SYCP3 (red). White arrow points to a Huwe1 + telomere end, yellow arrowhead points to the Huwe1 + sex body. Scale bar = 20 μm. (**b**) The Spo11-Cre KO animals have normal spermatogenesis. Hematoxylin and eosin stained testicular tissue sections from adult WT and Spo11-Cre KO. The yellow arrowheads indicate spermatids in the lumen of the seminiferous tubules. Scale bar = 90 μm (top panel). (**c**) The Spo11-Cre KO mice show the retention of γH2AX on the autosomes after homologous recombination. Representative immunofluorescence images of chromosome spreads prepared from WT and Spo11-Cre KO testes at 28 dpp stained for γH2AX (green) and SYCP3 (red) (left panel). White arrow indicates γH2AX foci on an autosome of a pachytene spermatocyte, an abnormality significantly more common in the Spo11-Cre KO testes. Quantification of different cell types from the images and of retention of γH2AX (right panel) WT, n = 4 (402 spreads), Spo11-Cre KO, n = 6 (613 spreads). (**d**) Spo11-Cre KO of Huwe1 did not lead to increased apoptosis. Shown are typical images from TUNEL assay performed on adult testes sections of WT and Spo11-Cre KO testes (left panel). Scale bar = 90 μm. Quantification from images (right panel) WT, n = 4 (335 tubules), KO, n = 4 (466 tubules). (**e**) RNA polymerase II remains excluded from the XY body of the Spo11-Cre KO spermatocytes. Representative immunofluorescence images of chromosome spreads prepared from WT and Spo11-Cre KO testes at 28 dpp stained for γH2AX (red) and RNA polymerase II (green) (n = 5). The white arrows indicate the absence of RNA polymerase II stain on the XY body. Scale bar = 20 μm. (**f**) Normal meiotic sex chromosome inactivation (MSCI) in the Spo11-Cre KO mice. Q-PCR measurements of mRNA levels of selected X-linked and Y-linked genes (n = 5). Data are represented as mean ± SEM. Student’s t-test. *p < 0.05.

Huwe1 is highly expressed in pachytene spermatocytes^30^. Interestingly, when we stained chromosome spreads of meiotic cells from the WT testis with anti-Huwe1 antibody, we found that Huwe1 was localized to the telomere ends of autosomes of pachytene spermatocytes and to the sex body, both regions of gene silencing (Fig. 5a). Histone H2A ubiquitination has been suggested to be important for meiotic sex chromosome inactivation (MSCI)^31–33^, and so we hypothesized that Huwe1 may regulate H2A and therefore be important for meiotic progression by regulating events such as MSCI in the pachytene spermatocytes. As we observed drastic loss of pre-meiotic germ cells with our previous models, we crossed hemizygous *Spo11-Cre* males with *Huwe1* ^*flox/flox*^ females to inactivate Huwe1 in spermatocytes that have initiated meiosis^34^. Driven by *Spo11* promoter, the Cre recombinase expression begins at 7 dpp in the leptotene spermatocytes^34^. Excision of the lox-P flanked region was confirmed by RT-PCR of whole testes RNA (Supplementary Figure 2). A fertility assay comparing the two genotypes revealed that the WT and *Huwe* ^*−/Y*^ *Spo11-Cre* (hereafter referred to as the Spo11-Cre KO) males had normal fertility (Supplementary Table S1). The histology of the testis of the Spo11-Cre KO was similar to that of the WT with no apparent defects in spermatogenesis as indicated by the presence of spermatids in the lumen of the seminiferous tubules (Fig. 5b). The epididymal sperm number and motility in the Spo11-Cre KO were normal (Supplementary Figure 5). To study meiotic progression more precisely, we prepared dissociated cell spreads from testes at 28 dpp. The spreads were stained with antibody to SYCP3, a component of the axial element which gives rise to the lateral element of the synaptonemal complex between homologous chromosome pairs, and with anti-γH2AX antibody, which labels the double strand breaks in leptotene and zygotene and the sex body in pachytene and diplotene spermatocytes^35^. Quantification of the different cell types did not reveal any significant defect in meiotic progression in the Spo11-Cre KO (Fig. 5c). Interestingly, the Spo11-Cre KO testis had a significantly higher percentage of spermatocytes that retained γH2AX on the autosomes in the pachytene and diplotene spermatocytes (where normally γH2AX is found only on the XY body) (Fig. 5c). However, this accumulation did not appear to lead to higher levels of apoptosis (Fig. 5d).

To explore if Huwe1 plays a role in MSCI, we studied the exclusion of transcriptional machinery from the XY body, one of the hallmarks of the process^36^. RNA polymerase II staining was absent from the XY body in the Spo11-Cre KO as in the WT indicating normal decrease in transcription on the sex chromosomes (Fig. 5e). To further explore for effects of Huwe1 deficiency on MSCI, we examined the expression levels of several sex-linked genes (*Ube1x*, *Atp7A*, *Gla*, *Chic1* on the X chromosome and *Ube1y*, *Rbmy*, *Zfy1*, *Zfy2* on the Y chromosome) (Fig. 5f). None were significantly altered between the two groups confirming that Huwe1 does not play an essential role in MSCI. Thus, the inactivation of Huwe1 in spermatocytes does not have significant consequences on the process of meiosis.

To further confirm that Huwe1 is not important in post-meiotic development during spermatogenesis, we crossed hemizygous *Prm-Cre* males with *Huwe1* ^*flox/flox*^ females to inactivate Huwe1 in the haploid spermatids^37,38^. We observed no significant differences in the sperm number and motility, and testis weights between the WT and Prm-Cre KO mice (data not shown). Histological evaluation of the testes sections revealed no evidence of impaired spermatogenesis in the Prm-Cre KO (data not shown). Thus, we can conclude that Huwe1 does not play an essential role in post-meiotic development during spermatogenesis.

## Discussion

In this report, we have inactivated the Huwe1 gene at key stages during spermatogenesis to conduct a thorough evaluation of the roles of this ubiquitin ligase in the differentiation of male germ cells. For the first time, we have shown that Huwe1 plays a critical role in the early steps of spermatogonial differentiation and entry into meiosis. Activating *Cre* recombinase in differentiating spermatogonia using the *Stra8* promoter resulted in degeneration of these cells as well as impaired replication and formation of pre-leptotene spermatocytes (Figs 2 and 3). However, Huwe1 is dispensable for the completion of meiosis and for spermatid maturation since activating Cre recombinase in leptotene spermatocytes or elongating spermatids using the *Spo11* or *Prm* promoters respectively had no effects on spermatogenesis or fertility (Fig. 5 and data not shown).

The depletion of differentiating spermatogonia and preleptotene cells upon loss of Huwe1 was associated with a hyperactivation of the DDR, revealed by the presence of intense foci of accumulation of γH2AX in these germ cells. Although we previously observed a similar accumulation of γH2AX in spermatogonial progenitors when Huwe1 was inactivated in gonocytes^18^, there are several significant differences between the two conditions. Most importantly, the triggering of the DDR in the differentiating cells did not lead to mitotic catastrophe as we observed in the progenitors, but instead led to death by apoptosis (Fig. 4). In addition, mechanisms downstream of recruitment of γH2AX to the sites of DNA damage were impaired in differentiating spermatogonia lacking Huwe1. Accumulation of ubiquitin in the γH2AX containing foci was noticeably absent (Supplementary Figure 3). This contrasts with the normal accumulation of ubiquitinated proteins to γH2AX foci in spermatogonial progenitors when Huwe1 was inactivated in gonocytes^18^. Thus, this defect in the downstream DDR may have led to excessive activation of the upstream signaling and activation of apoptosis. At this time, we cannot rule out other possible explanations for the different responses. For example, it remains possible that the spermatogonial progenitors and differentiating spermatogonia express different activities of their apoptotic machinery with progenitors having a less active or incomplete machinery and therefore unable to couple the DDR to an apoptotic pathway. Indeed, the mitotically quiescent SSCs have been shown to be more resistant to low doses of irradiation compared to differentiating spermatogonia, also suggesting a tighter coupling of signaling in differentiating spermatogonia that leads to apoptosis^39^. Further studies will be required to delineate the mechanisms more precisely.

Interestingly, although inactivation of Huwe1 in leptotene spermatocytes did not exert any evident effect on spermatogenesis, it still led to accumulation of γH2AX (Fig. 5). This accumulation was less marked than that observed in differentiating spermatogonia and was found on autosomes in pachytene and early diplotene spermatocytes, sites of double strand breaks induced during meiotic recombination. This was not associated with any apparent defect in spermatocyte development and would be consistent with the notion that the degree of hyperactivation of the DDR may be important in determining the cellular response. The modest hyperactivation seen here may have allowed resolution of the DDR in contrast to the extreme hyperactivation seen when Huwe1 was inactivated in less differentiated cells.

We found that inactivation of Huwe1 at three different developmental time points – gonoctyes, differentiating spermatogonia and spermatoctyes - led to accumulation of γH2AX. We previously showed that inactivation of Huwe1 in primary spermatogonia led to accumulation of also the non-phosphorylated precursor H2AX and that the accumulation of γH2AX is due, at least in part, to the increased availability of the precursor protein. All of this is consistent with Huwe1 targeting H2AX for ubiquitination and degradation as has been demonstrated in fibroblasts^40^. It is possible that other ubiquitin ligases exist for H2AX or γH2AX and may be active in spermatoctyes, thereby limiting the increase in γH2AX seen upon loss of Huwe1 in these cells. Such alternative, redundant mechanisms for degrading H2AX, γH2AX could also explain the lack of a significant phenotype upon inactivation of Huwe1 in meiosis or spermiogenesis.

Huwe1 is a large protein having a wide array of substrates that have been characterized at a cellular level, but the physiological functions of Huwe1 *in vivo* remain poorly characterized. Of the limited functions currently known, several of these involve modulation of tissue specific stem cells. In both neural^41^ and hematopoietic^42^ development, Huwe1 suppresses proliferation of stem cells by targeting N-myc, a vital transcriptional regulator that promotes progression through the cell cycle. Loss of Huwe1 in these tissues promotes proliferation, differentiation and exhaustion of the stem cell pool. In contrast, our current results together with those of our recent report^18^ indicate an opposite effect of loss of Huwe1 on cell growth and instead identify novel, critical roles for Huwe1 in the maintenance and differentiation of spermatogonial stem cells and associated progenitors. These roles are distinct from those in the neural and hematopoietic lineages as Huwe1 appears to target a different substrate and the loss of Huwe1 results not in hyperproliferation, but in impaired cell division due to hyperactivation of the DDR. Thus, Huwe1 performs vital functions in modulating DNA repair and thereby plays a critical role in maintaining genomic stability during the proliferation of spermatogonia, which is essential for normal spermatogenesis.

## Methods

All procedures were carried out in accordance with the regulations of the Canadian Council for Animal Care and were approved by the Animal Care Committee of the McGill University Health Centre Research Institute.

### Animals

Conditional Huwe1 knockout (*Huwe1* ^*flox/flox*^) mice were kindly provided by Drs. Antonio Iavarone and Anna Lasorella^41,43^. To inactivate Huwe1 specifically in the differentiating spermatogonia, spermatocytes or spermatids, *Huwe1* ^*flox/flox*^ females were bred with male mice hemizygous for *Stra8-Cre* (Tg(Stra8-Cre)1Reb)^19^ (Jackson Laboratory), *Spo11-Cre* ^20^ (kindly provided by Dr. Paula Cohen) or *Prm-Cre* ^37^ (Jackson Laboratory) respectively. *Huwe1* ^*flox/Y*^ (WT), *Huwe1* ^*−/Y*^ *Stra8-Cre*, *Huwe1* ^*−/Y*^ *Spo11-Cre*, and *Huwe1* ^*−/Y*^ *Prm-Cre* (KO) male offspring were identified by genomic PCR on tail DNA using oligonucleotides derived from the *Cre* recombinase sequence (Table 1).

**Table 1:** Oligonucleotide information.

Gene name		Experiment	Sequence 5′ - 3′
Stra8	F	QPCR	CATCATCACTGGGTTGGTTG
	R	QPCR	CTGCGTGTTCCACAAGTGTC
Dazl	F	QPCR	TCCTTGACTTGTGGTTGCTG
	R	QPCR	CCACCTTCGAGGTTTTACCA
Sohlh2	F	QPCR	CCGTGCTTTTGGCTGCTAAC
	R	QPCR	TCACTGAAACTAATGTCAGCTCC
Spo11	F	QPCR	TGCTTTGTCAGTGGGATCAA
	R	QPCR	TACGGATCCATGTCCATGTC
Smc1b	F	QPCR	CATGAGGGAAAACGTCAGCAG
	R	QPCR	TGACACAGATCAAGCAGTCTTC
Sycp1	F	QPCR	TGTCCAGTCGGGAAAACATTG
	R	QPCR	AGAATTAGCAACCTGTTCGAGC
Sycp3	F	QPCR	AGCCAGTAACCAGAAAATTGAGC
	R	QPCR	CCACTGCTGCAACACATTCATA
Cre Spo11/Prm-Cre)	F	PCR	CCAGGCTAAGTGCCTTCTCTACA
	R	PCR	AATGCTTCTGTCCGTTTGCCGGT
Cre (Stra8-Cre)	F	PCR	GTGCAAGCTGAACAACAGGA
	R	PCR	AGGGACACAGCATTGGAGTC
Huwe1	F	Genotyping RT-PCR	GGCCTAGTTTATCTGCTTG
	R	Genotyping RT-PCR	GGATGGCCAATAGTAGCATG

To measure cell proliferation *in vivo*, mice were injected intraperitoneally with 50 μg/g BrdU in saline and sacrificed 24 hr later. Testes were isolated, fixed and stained as described below.

Synchronization of the seminiferous tubules was achieved as previously described^23^. Briefly, 2 dpp mice were pipette fed 100 μg/g bodyweight of WIN 18446 (Tocris Bioscience), an inhibitor of retinaldehyde dehydrogenase enzyme, which is required for retinoic acid synthesis), suspended in 1% gum tragacanth (Sigma-Aldrich Canada) for 7 consecutive days. As retinoic acid is essential for spermatogonial differentiation, this treatment maintained the spermatogonia in an undifferentiated state. To initiate synchronous differentiation in all the tubules, the mice were given a subcutaneous injection of 100 μg retinoic acid (Sigma-Aldrich #R2625, Canada) diluted in 10 μl of DMSO at 9 dpp. Animals were sacrificed at the indicated times.

### Analysis of sperm number and motility

For determination of sperm number, caudae epididymes were isolated from WT and KO mice, poked with a 27-gauge needle and placed in 2 ml of pre-warmed Hanks medium M199 (Invitrogen Canada Inc.). The spermatozoa were allowed to swim out for 10 min on a small plate maintained at 37 °C. Sperm were counted using a hemocytometer. For analysis of sperm motility, caudae epididymes were treated as above, but incubated in media supplemented with 3 mg/ml BSA^44^. Total and progressive sperm motility were evaluated at 37 °C using a computer-assisted sperm analysis system (CASA) with Sperm Vision HR software version 1.01 (Minitube, Ingersoll, ON, Canada). For analysis of motility, a total of 200 spermatozoa were examined for each sample and the percentages of sperm total and progressive motility were analyzed.

### Preparation of chromosome spreads

Glassware was washed thoroughly with ammonium hydroxide solution (Windex) followed by warm tap water and double distilled water to remove any contamination of grease. Chromosome spreads for immunofluorescence staining were obtained from 28-day old mice, a time at which the WT mice were expected to have completed meiosis in the first wave of spermatogenesis. Spreads were prepared as described^45^ with modifications. The testes were isolated in MEM media with Hanks salts (MEMH) and their tunica removed. The tubules were rinsed in MEMH in a petri dish and minced with a razor blade. They were then squeezed with a pair of forceps following which they were transferred to a microfuge tube and allowed to stand in MEMH media. The media was aspirated away from the tissue and spun at 300 g for 3 min. The supernatant was removed and the cell pellet gently resuspended in residual MEMH. The cell suspension (2–3 μl) was expelled through a 20 μl micropipette on to the convex surface of 0.5% NaCl solution (completely filling a 60 mm petri dish) and allowed to spread. Following this, the spread cells were picked up by lowering a glass slide flat on the solution surface for 10 sec and fixed by placing the slide in 2% paraformaldehyde. The slides were washed in 0.4% Photo-Flo solution, dried at room temperature and stained as described below.

### Tissue preparation, histology, immunostaining and TUNEL assay

Testes were fixed in 4% paraformaldehyde. For histological analysis, 4 μm sections were prepared from paraffin-embedded testes samples and stained with hematoxylin and eosin. For immunofluorescence staining, the sections were rehydrated followed by antigen retrieval performed by microwaving in 10 mM citrate buffer (pH 6.0) for 6 min (maximum power). The slides were allowed to stand in the buffer for 2 min and then microwaved again for 10 min (60% power). Sections were then blocked using 10% goat serum and 1% BSA. Primary antibody (Table 2) incubation was carried out at 4 °C overnight in PBS containing 1% BSA, 2% goat serum and 0.02% Triton X-100. The next day, the sections were incubated for 2–4 hrs with appropriate secondary antibodies diluted in PBS with 1% BSA and 0.01% Triton X-100. For immunofluorescence staining of chromosome spreads, the slides were stained using the above protocol excluding the steps of rehydration and antigen retrieval. Sections were counterstained with Hoechst (Sigma, H6024), mounted with ProLong Gold antifade (Life Technologies, P36930) and examined using a Zeiss Axiovert 200 microscope. Metamorph software (version 6.3r7) was used to capture the images. Immunohistochemical staining was performed using the Vectastain ABC kit. TUNEL assay was carried out using the ApopTag Plus Peroxidase *In Situ* Apoptosis Detection Kit (Millipore, S7101). The slides were mounted with Clear-Mount (Electron Microscopy Sciences, 17985–12) and images captured on a Zeiss Axioscop 2 microscope using AxioVision Rel. 4.8 software. The slides were coded to blind the observer to the experimental condition during quantification.

**Table 2:** Antibody information.

Protein name	Company	Catalog #	Conc. for immunostaining
Stra8	Dr. Griswold, Washington State University	NA	1:2000
BrdU	Roche	11 170 376 001	1:50
γH2Ax	Abcam	ab11174	1:2000
γ-H2Ax	EMD Millipore	05–636	1:500
Sycp3	Abcam	ab15093	1:100
FK2	EMD Millipore	04–263	1:100
pCHK2	Cell Signaling Technology	2661 S	1:100
Cleaved Caspase 3	Cell Signaling Technology	9661	1:100
p53 (phospho S15)	Abcam	ab1431	1:50

### RT-PCR and quantitative PCR

Total RNA was extracted from mouse testes using TRIzol reagent (Invitrogen). cDNA was synthesized from 1 μg of RNA using the High Capacity cDNA Reverse Transcription Kit (Applied Biosystems). Primers used for RT-PCR and qPCR are shown in Table 1. QPCR was performed using *Power* SYBR® green PCR master mix in a ViiA7 qPCR analyzer (Applied Biosystems). Expression of genes was normalized to GAPDH. Differential expression was represented as fold change (2^−ΔΔCT^).

### Statistical analysis

All statistical analysis was carried out using GraphPad Prism version 6.0 (GraphPad Software). The unpaired Student *t-*test was used to analyze the results. P < 0.05 was considered as significant. The numbers of animals and tubules/chromosome spreads assessed per animal have been indicated in the figure legends.

### Data Availability

All data that has not been included in this research article will be made available upon request to the corresponding author.

## Electronic supplementary material

Supplementary Figures and Table
