# Supplementary material for: Ubiquitin Ligase Huwe1 Modulates Spermatogenesis by Regulating Spermatogonial Differentiation and Entry into Meiosis
Source: Sci Rep. 2017 Dec 19;7:17759. doi: 10.1038/s41598-017-17902-0 (PMC5736635; doi:10.1038/s41598-017-17902-0)
Supplement: Supplementary file 1 — Supplementary Figures and Table [file 41598_2017_17902_MOESM1_ESM.pdf]

Ubiquitin Ligase Huwe1 Modulates Spermatogenesis by Regulating Spermatogonial Differentiation and Entry into Meiosis

Bose R, Sheng K, Moawad AR, Manku G, O'Flaherty C, Taketo T, Culty M, Fok KL, Wing SS

Supplementary Figures and Table

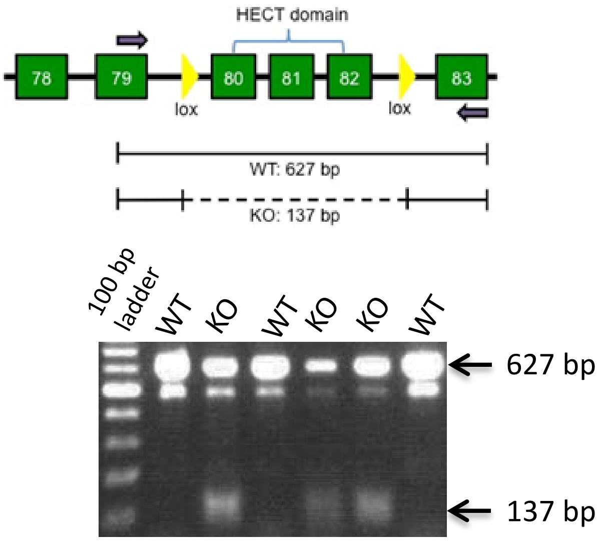

Supplementary Figure 1. Huwe1 is inactivated in the testis using Cre-recombinase driven by the Stra8 promoter. Schematic representation of the location of the oligonucleotides used for testing the recombination. Arrows indicate forward and reverse oligonucleotides located in exons 79 and 83 respectively. Indicated are the expected band sizes from WT and Stra8-Cre KO testis after RT-PCR (top panel). Representative image of the gel with the RT-PCR products (bottom panel).

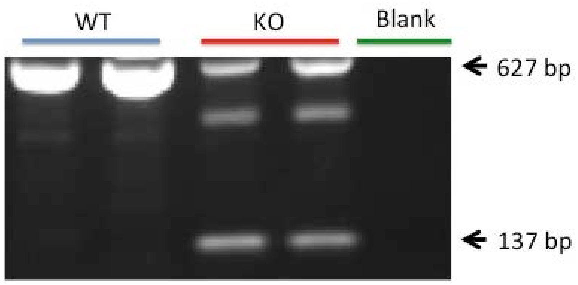

Supplementary Figure 2. Huwe1 is inactivated in the testis using Cre-recombinase driven by the Spo11 promoter. Oligonucleotides used for testing the recombination have been described in Supplementary Figure 1. Representative image of the gel with the RT-PCR products.

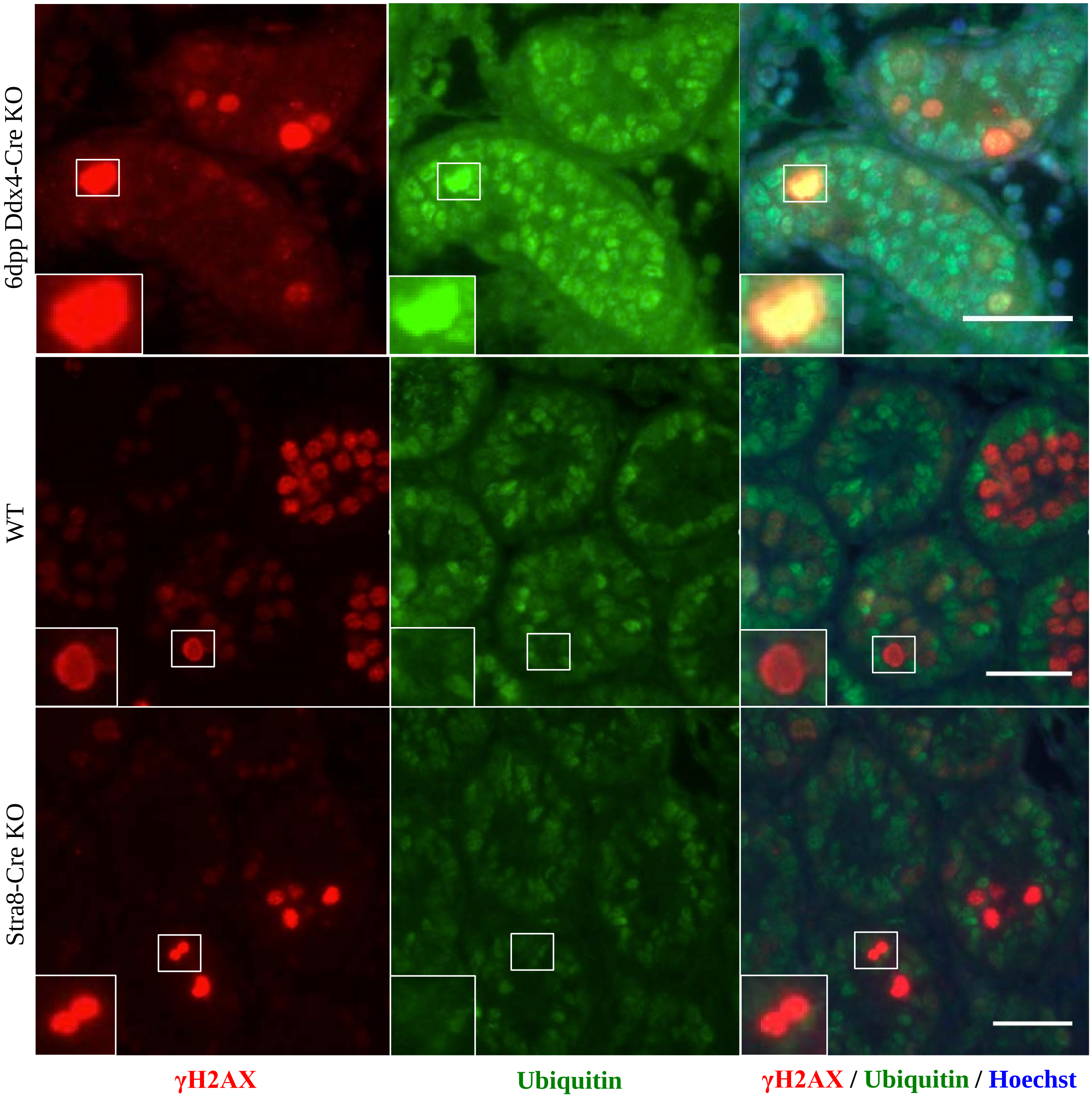

Supplementary Figure 3. Inactivation of Huwe1 in the differentiating spermatogonia leads to impaired downstream signaling of the DDR. The Stra8-Cre KO mice do not show significant foci of ubiquitin at the intense γH2AX foci. Representative images from 10 dpp WT and Stra8-Cre KO testes stained with antibodies for γH2AX (red), ubiquitin (FK2, green) and Hoechst (blue) (n=3). Also shown are images of testis sections from 6 dpp mice in whom Huwe1 had been inactivated using the Ddx4-Cre promoter, used as a positive control for observing the activation of DDR downstream of γH2AX. Scale bar = 20 μm.

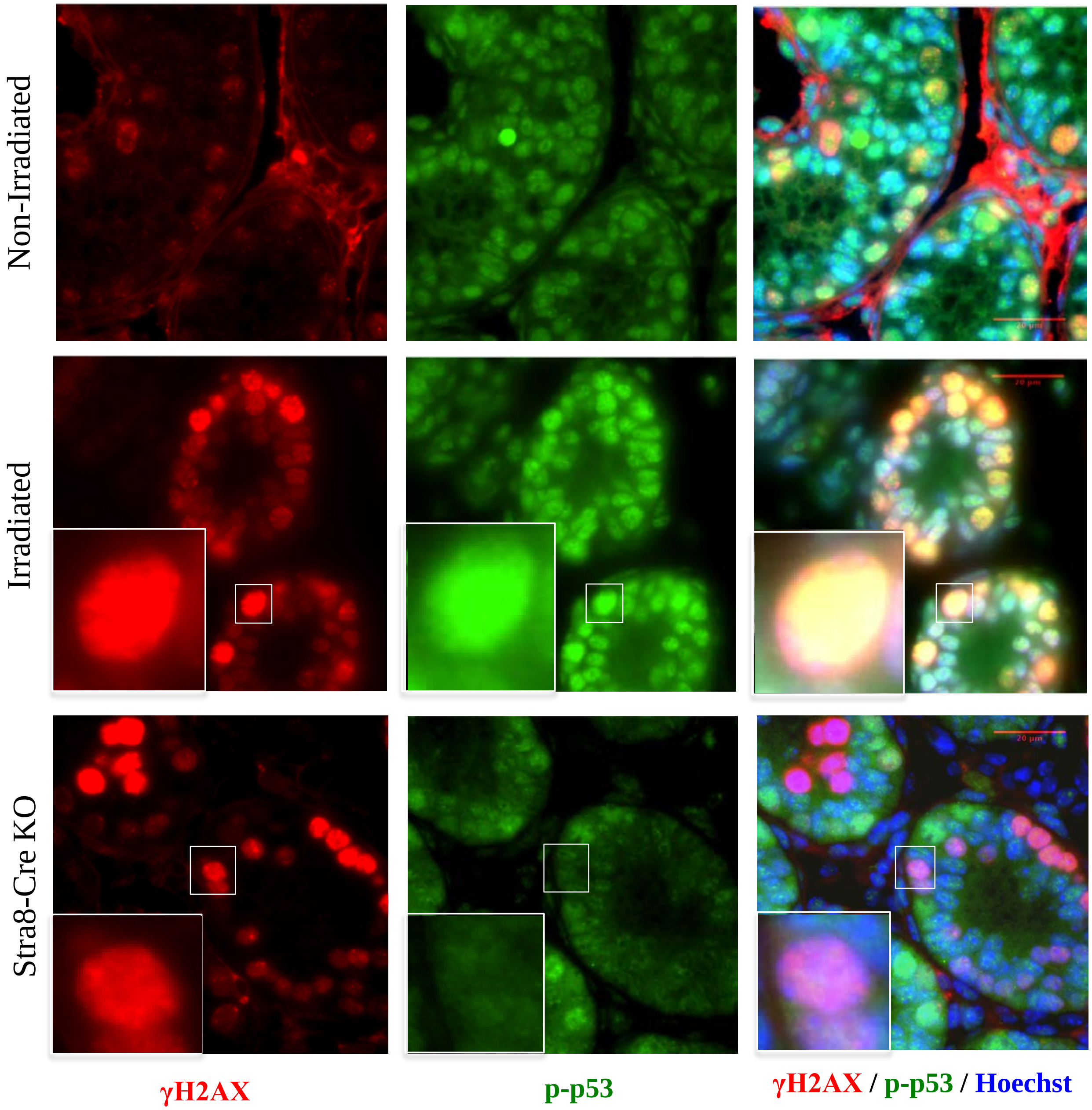

Supplementary Figure 4. Hyperactivation of the DNA damage response in germ cells of Stra8-Cre KO mice is not associated with activation of p53. Testis sections of Stra8-Cre KO mice were stained with antibodies to γH2AX (red), phosphorylated p53 (green) and Hoechst (blue). As a positive control, normal testis was irradiated with 10 Gy and isolated 30 minutes later and compared to non-irradiated control testis. Scale bar = 20 μm.

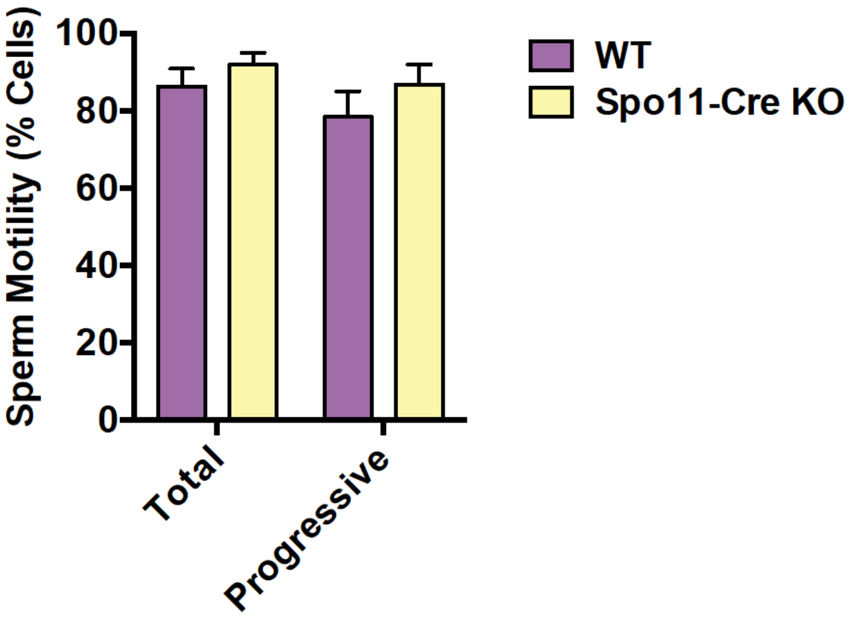

Supplementary Figure 5. Inactivation of Huwe1 in spermatocytes does not affect sperm motility
Sperm motility analysis in WT (n=4) and Spo11-Cre KO (n=5) mice using CASA.

	#Matings	#Litters	#Pups	Average Litter Size
WT	12	10	123	12
Spo11-Cre KO	12	10	112	10

Supplementary Table 1. Inactivation of Huwe1 in the meiotic spermatocytes did not affect fertility

WT and Spo11-Cre KO male mice (n=3) were mated with CD1 females for 4 consecutive weeks. The females were changed every week. Indicated are the number of litters sired and the average litter size.
